# Reinforcement-Learning-Based Route Generation for Heavy-Traffic Autonomous Mobile Robot Systems

**DOI:** 10.3390/s21144809

**Published:** 2021-07-14

**Authors:** Dominik Kozjek, Andreja Malus, Rok Vrabič

**Affiliations:** Faculty of Mechanical Engineering, University of Ljubljana, SI-1000 Ljubljana, Slovenia; dominik.kozjek@fs.uni-lj.si (D.K.); andreja.malus@fs.uni-lj.si (A.M.)

**Keywords:** intralogistics, autonomous mobile robots, multi-robot cooperation, reinforcement learning, route planning

## Abstract

Autonomous mobile robots (AMRs) are increasingly used in modern intralogistics systems as complexity and performance requirements become more stringent. One way to increase performance is to improve the operation and cooperation of multiple robots in their shared environment. The paper addresses these problems with a method for off-line route planning and on-line route execution. In the proposed approach, pre-computation of routes for frequent pick-up and drop-off locations limits the movements of AMRs to avoid conflict situations between them. The paper proposes a reinforcement learning approach where an agent builds the routes on a given layout while being rewarded according to different criteria based on the desired characteristics of the system. The results show that the proposed approach performs better in terms of throughput and reliability than the commonly used shortest-path-based approach for a large number of AMRs operating in the system. The use of the proposed approach is recommended when the need for high throughput requires the operation of a relatively large number of AMRs in relation to the size of the space in which the robots operate.

## 1. Introduction

Autonomous mobile robots (AMRs) are increasingly being used in modern intralogistics systems instead of autonomously guided vehicles (AGVs). They are often used when additional flexibility is required in cargo transportation tasks, since AMRs, unlike AGVs, are capable of moving in free space without additional floor markings such as painted lines or magnetic tapes. Even though they can move freely, it is often necessary to restrict the areas in which they are allowed to move in order to improve the safety and efficiency of their operation. For example, they are often restricted to move along walls or within predefined routes to improve system safety. Another example is movement in a narrow corridor, where it makes sense to implement a one-way system; otherwise, deadlocks might occur when vehicles moving in opposite directions encounter each other. Designing movement constraints and rules, e.g., in the form of predefined routes, is a difficult problem because it is layout and case specific. Nevertheless, by designing sensible routes, system efficiency can be improved.

Planning a movement trajectory is done by algorithmically searching through the representation of the space and finding the optimal solution based on a desired criterion, which is often the length of the path. Common approaches to finding a path for a robot are to use one of the basic path planning algorithms, such as A* [1], probabilistic roadmap planner (PRM) [2], rapidly exploring random trees (RRT) [3], and potential fields algorithms [4] or their extensions.

When multiple robots share the same environment, the problem of finding the optimal solution for all robots cannot be solved in polynomial time. Therefore, motion planning is often treated separately from robot coordination. For multiple robots to move in the same environment, the approaches to planning a path for moving from one point to another can be divided into two main groups. The movement for the robots is planned by either considering one robot at a time or multiple robots. In the first case, the resolution of potential conflicts is handled on-line during movement execution [5] while the second approach detects and prevents some of the conflicts off-line in advance.

In Erdmann [6], the planner determines the path for each robot one by one according to a priority order, where each already planned path is considered when planning the path for the next robot in each case. Pecora et al. [7] solve conflicts on paths of multiple vehicles by progressively defining trajectories using several constraint solvers, which guarantees their conflict-free execution.

AGVs usually move along predefined floor markings. The markings represent the roads for the AGVs. They restrict possible interactions between AGVs to intersections and prevent collisions and path crossings in other areas. The roadmaps are usually defined by a human expert; however, some solutions for automatic roadmap generation can also be found in the literature. In Uttendorf [8], the Bellman–Ford algorithm, the A* search algorithm, and fuzzy inference logic are subsequently applied in the roadmap generation process. The approach of Digani [9] considers coverage, connectivity, and redundancy of paths in computing a roadmap for the AGVs.

There are examples of using the approach of roadmaps for free-ranging robots. In Kleiner [10], linear programming is used to construct a roadmap for path planning from a Voronoi graph, which is adapted in real time to the detected change in the environment. The idea of Henkel [11] is to generate a roadmap that is a directed graph optimised for collision and deadlock avoidance that can be used in centralised and distributed planning setups. For complete collision prevention, the approach still assumes negotiation at run-time.

Large solution spaces of planning problems often prevent the design of exact algorithmic solutions and keep the problems open to the application of new ideas to solve them. Several research papers present approaches that use artificial intelligence (AI) methods, such as machine learning (ML), which are making progress in their ability to solve many real-world problems. Robot motion planning is addressed by data-driven ML approaches to varying degrees; from learning that replaces the entire navigation stack to learning that is only used in navigation subsystems or in components within the navigation stack [12]. Reinforcement learning (RL), a type of ML technique, is the most commonly used. In [13], an RL agent performs the functionality of a local planner for a conventional robotics procedure using the PRM planner. A similar problem solution is used in [14], where the focus is on incremental training of the RL agent.

For settings where multiple robots share their movement space, RL approaches are used in a multi-agent form. In both [15,16], multiple RL agents simultaneously plan their local paths in a distributed setting. The first research incorporates expert knowledge into the learning phase by using imitation learning (IL), while the second improves the convergence to the optimal policy using an evolutionary training approach.

The paper addresses the problems of off-line route planning and on-line route execution. Given a known layout, the goal is to improve the efficiency and the scalability of an AMR-based intralogistics system by designing a system of routes between pick-up and drop-off locations. On the one hand, the routes should be short, and on the other, they should intersect as little as possible, since intersections are sources of lost efficiency due to vehicle encounters requiring evasive manoeuvres and potential deadlocks. Computationally, this is an extremely challenging problem, since, in addition to finding possible routes between pick-up and drop-off locations, it is also necessary to check how each route intersects with the others. Combinatorial complexity makes it impossible to tackle this problem with an exhaustive search. Instead, a reinforcement learning approach is proposed, in which an agent is trained to generate routes on a given layout while aiming to satisfy various criteria to achieve the desired properties of the generated route map. The agent learns to satisfy the criteria by improving its policy based on the rewards received in the training phase.

The proposed approach is evaluated by observing the execution of a list of transport tasks for a given shop floor layout. A comparison is made between the AMR transport system using the route map generated by the RL agent to the same transport system generating paths on the fly using A*. The comparisons are made for AMR fleets of different sizes. The results show that the proposed approach performs better in terms of throughput and reliability than the commonly used shortest path-based approach in systems with a higher number of AMRs. Compared to the traditional route planning methods (such as A* search algorithm, RRT, etc.), the advantages of the proposed method are scalability (the number of AMRs in the system does not affect the difficulty and time of route computation), minimisation of possible interactions between AMRs (this increases the robustness of the system), and the need for communication between AMRs in the route execution phase is minimal (the mobile robots only need to exchange their current position and orientation). The disadvantages are that the computational complexity increases with the number of routes to be found, pick-up/drop-off locations must be determined in advance, and generated routes are static.

## 2. Reinforcement-Learning-Based Route Generation

**Figure 1:**
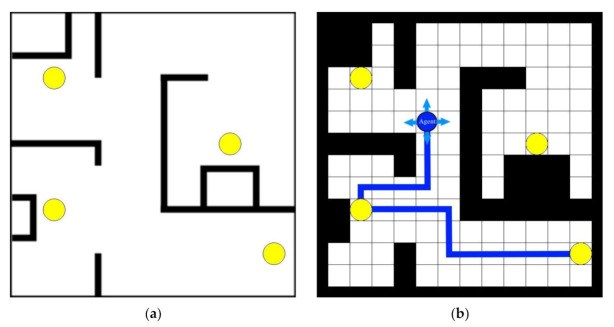
Input layout and the corresponding occupancy grid: (**a**) layout with pick-up/drop-off locations (yellow circles), white represents the driving area and black are the walls or other static obstacles; (**b**) the corresponding occupancy grid with the reinforcement-learning agent searching for the routes between pick-up and drop-off locations (the light blue arrows around the agent illustrate four possible actions).

**Figure 2:**
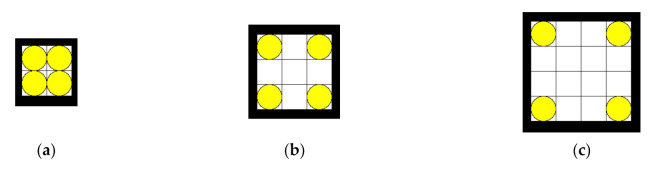
Example layouts to demonstrate computational complexity: (**a**) 2 × 2, four pick-up/drop-off locations (four pick-up/drop-off locations means there are 12 directed routes between pick-up/drop-off locations to be found); (**b**) 3 × 3, four pick-up/drop-off locations; (**c**) 4 × 4, four pick-up/drop-off locations. The transition between cells is only in directions up, down, left, and right).

The goal of the proposed method is to automatically generate directed routes between pick-up and drop-off locations for a given layout such that the routes are as short as possible, the mobile robots do not travel in the opposite direction, and there are as few intersections and turns as possible. The input data is the layout, which determines static obstacles and locations of the pick-up/drop-off locations (see Figure 1a). If there are *N* pick-up/drop-off locations and all of them are considered as both pick-up and drop-off locations, then the number of directed routes between them is *N·*(*N − 1*). If there is an optimisation function that indicates the quality of a particular combination of routes, the brute force approach to finding the best combination of these routes would be to compute the value of the optimisation function for each combination and select the one with the highest value. To demonstrate the computational complexity, some simulation results are shown in Table 1: (1) the total number of different directed route combinations, and (2) the estimated times to find all combinations and compute the values of the optimization function for all route combinations for three simple example layouts shown in Figure 2 using exhaustive search. Due to the extremely large solution space, such an exhaustive search is computationally too expensive, so a heuristic search is required.

The artificial intelligence technique called reinforcement learning is used to find a good combination of routes. The idea is to use a reinforcement learning (RL) agent to generate routes between pick-up and drop-off locations. To enable discrete actions of the RL agent and thus make it easier for it to search for routes, the first step is to generate an occupancy grid based on a given layout (Figure 1b). The cell size must be larger than the largest dimension (length or width) of the mobile robot. This is to allow the robots to drive and turn without interfering with the robots in the neighbouring cells. At each step, the RL agent extends the route by one cell. To do that, it can choose one of four possible actions: (1) a move up, (2) a move left, (3) a move right, or (4) a move down. Figure 1b shows an agent that has already generate a possible route between the pick-up location at the bottom right and the drop-off location at the bottom left and is now generating a second route starting from the pick-up location at the bottom left.

During the learning process, the RL agent repeatedly attempts to generate a route map containing all routes between pick-up and drop-off locations. Each attempt is a sequence of steps called an episode. The routes are determined sequentially in random order. An episode ends when all routes have been determined or all attempts to find the routes have been completed.

During each learning step, the agent performs an action, makes an observation and receives a reward. At each step, the agent has information about its current location on the occupancy grid, the pick-up and drop-off locations of the current route, and can detect obstacles and other pick-up/drop-off locations in the neighbouring cells of the occupancy grid. After movement, the previous location of the agent is stored among the already visited locations. The state of the environment (***s***) is represented by the position of the current pick-up location (***p****^pickup^* ), the position of the current drop-off location (***p****^dropoff^* ), the current position of the agent (***p****^agent^* ), the adjacency of walls or other static obstacles around the agent (***w***), whether the neighbouring cells have already been visited in the current episode (***v***) or during the current route search (***v****^cr^*), and adjacency of other (excluding current drop-off location) pick-up/drop-off locations (***o***):

***s*** = (***p**^pickup^*, ***p**^dropoff^*, ***p**^agent^*, ***w***, ***v***, ***v**^cr^*,***o***) (1)

Positions of the current pick-up, drop-off, and agent locations (***p****^pickup^*, ***p****^dropoff^*, ***p****^agent^*) are defined as:

***p**^pickup^* = [*x*, *y*] (2)

where *x* is the coordinate in the x-direction, and *y* is the coordinate in the y-direction. The same applies to ***p****^dropoff^* and ***p****^agent^*.

The adjacency of walls, other static obstacles, already visited locations, and other pick-up/drop-off locations (***w***, ***v***, ***v****^cr^*, and ***o***) are sets of four binary elements that tell the agent where walls, other static obstacles, already visited locations, or other pick-up/drop-off locations are around it. Adjacency is defined for the case of walls and other static obstacles as:

***w*** = [*w_up_*, *w_down_*, *w_left_*, *w_right_*] (3)

where each of the four elements (*w_up_*, *w_down_*, *w_left_*, *w_right_*) has the value of either 1 or 0, depending on whether a wall or other static obstacle is above, below, to the left, or to the right of the agent (in which case the value is 1) or not (in which case the value is 0). The same applies to the other three adjacency sets ***v***, ***v****^cr^*, and ***o***.

The agent learns to choose its actions according to the desired goals by receiving appropriate rewards for its behaviour in the environment. While reinforcement learning often uses simple and sparse rewards, a reward composed of multiple components allows for better adaptation of the learned policy to specific goals of the path planning problem, as seen for example in [16,17]. Therefore, the reward function is designed to discourage the agent from generating routes that are longer than necessary, encourage one-way drives across a single cell, and keep the number of turns on each path and intersections between successive routes low. At each step, the agent receives a reward *r*:

*r* = *r_step_* + *r_sp_* + *r_position_* + *r_visited_* + *r_visit.c.r_* + *r_opp_* + *r_cross_* + *r_turn_* + *r_max.stp._* + *r_dropoff_* (4)

The values of the individual parts are calculated as shown in Table 2.

The agent receives a penalty *r_step_* (penalty because the value of *R_step_* is negative) for each step to force the agent to generate short routes. *r_sp_* is a reward or a penalty depending on whether the distance to the drop-off location computed as the shortest route has decreased or increased in the last step. This part of the reward function is intended to help the agent find drop-off locations faster. *r_position_* is a high penalty that the agent receives if it tries to go in a direction where it bumps into a wall or other static obstacle, or if it is at the wrong drop-off location. In this case, the agent is also randomly moved to one of the adjacent unoccupied cells. *r_visited_* is a penalty the agent receives if it is in a location that has already been visited in the current episode, and *r_visit.c.r._* is another high penalty the agent receives if it is in a location that has already been visited during the current route search. In general, the agent should not revisit locations on other routes when searching for a particular route, but in some cases, this either cannot be avoided or this solution is still better overall than the alternatives (e.g., finding a longer route to avoid revisiting locations). *r_opp_* is a penalty that the agent receives if any part of the currently generated route at the agent’s current location goes in the opposite direction than another part of the already existing routes. This is a high penalty because the occurrence of robots driving in the opposite direction at the same location should be prevented if possible. *r_cross_* is a penalty for crossing other existing routes (*n_cross_* is the number of routes the agent crosses), and *r_turn_* is a penalty for turning. *n_stp.c.r._* is the number of steps in the search for the current route, *N_stp.max_* is the predefined maximum number of steps allowed for a single route, and *r_max.stp._* is a penalty if the number of steps for the current route exceeds the predefined maximum number of steps allowed per route (in this case, the search for the current route is aborted, which means that the agent will not be able to find all routes in this current episode). The number of steps in route search is limited to avoid too long (or infinitely long) episodes, which is particularly important in some cases in the early stages of the learning process. Finally, *r_dropoff_* is a high reward that the agent receives when it arrives at the drop-off location of the current route. This is the part of the reward function that encourages the agent to find the route to the drop-off location. The more skillfully the agent avoids situations for which it would be punished by the punishing parts of the reward function, the higher the total reward that the agent is ultimately left with.

*R_step_*, *R_sp_*, *R_visited_*, *R_cross_*, and *R_turn_* are the constants: learning parameters that must be set before starting the learning process, and which can be used to set the relative importance of a single part of the reward function (e.g., in some cases route intersections might be more undesirable and route length is not as important, but there might be other cases for which route length is more important than the number of route intersections). These values depend on a specific application.

## 3. Experimental Validation

### 3.1. Case Study Description

The case study shows how the use of the proposed method can enable higher performance of a multi-AMR system than the commonly used shortest path-based approach. Performance is defined as the number of tasks completed per time:

*performance = tasks/time* (5)

where *tasks* is the number of tasks given to a multi-AMR system and *time* is the total time required to complete all tasks. A task is defined as the pair of a pick-up location and a drop-off location:

*task = (pickup_location_id, dropoff_location_id)* (6)

when a *task* is given to a mobile robot, the mobile robot must first travel to the pick-up location and then to the drop-off location. The task is finished when the mobile robot arrives at the drop-off location. Another aspect of performance is the scalability of a multi-AMR system, which could be defined as the number of robots in the system without serious conflicts between the robots that would prevent the completion of the given tasks.

The proposed method is compared with the baseline approach, which uses the shortest paths from pick-up to drop-off locations. To ensure the same conditions for both approaches, five schedules, each defining the list of ten tasks, are pre-generated in a random manner. Each of these five schedules is given to both (proposed and baseline) methods for a different number of AMRs in the system. The tests start with a single robot in the system, and then the number of robots is increased until the maximum number (10) is reached or the system faces severe conflicts between the robots that prevent the completion of the tasks.

**Figure 3:**
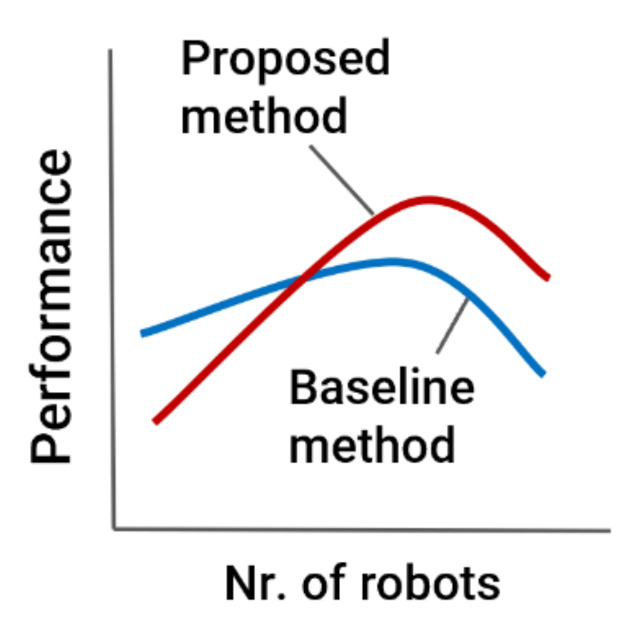
The expected result.

The expected result is that the shortest path based method (baseline) has higher performance when the number of mobile robots in a system is small because in the baseline approach, when the number of robots is small, conflicts between robots do not occur as frequently and the mobile robots take less time on average to complete the tasks due to shorter routes. The routes generated by the proposed approach are expected to be longer than the shortest paths because other criteria besides route length are used. When the number of mobile robots is larger, the proposed method is expected to have higher performance than the baseline method, since in this case, it becomes more important how well conflicts can be avoided since encounters with mobile robots are more frequent. Finally, when the number of mobile robots in a system is very large, the performance is expected to decrease in both cases (see Figure 3).

**Figure 4:**
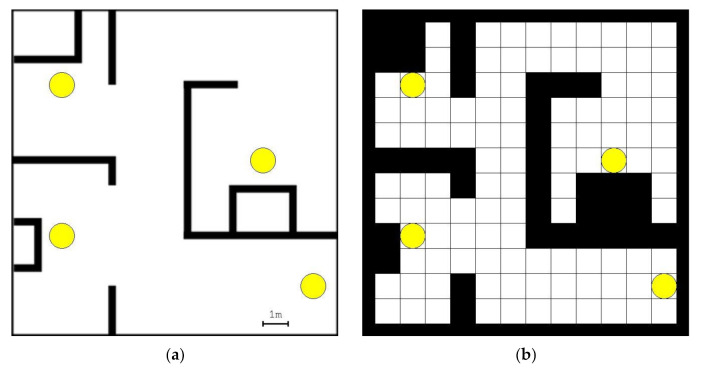
(**a**) Layout for the experiments and (**b**) the corresponding occupancy grid with the cell size of 1 m.

MiR100 autonomous mobile robots are used in the experiments. The main dimensions of MiR100 are length 890 mm, width 580 mm, and height 352 mm [18]. The layout for the experiments and the corresponding occupancy grid is shown in Figure 4. All four marked pick-up/drop-off locations are used both as pick-up and as drop-off locations, meaning that 12 routes have to be found.

### 3.2. Reinforcement-Learning Parameters

Besides the definition of the possible actions, the state of the environment, and the reward function, which have already been presented in Section 2, there are additional parameters of reinforcement learning that need to be set. For the experiments, the reinforcement learning algorithm proximal policy optimisation (PPO) reinforcement learning algorithm [19] was used. The implementation of the PPO algorithm (named PPO1) from the Stable Baselines programming library [20] was used. The total number of steps to train the RL agent was set to 5 million. One-hot encoding was used for ***p****^pickup^*, ***p****^dropoff^*, and ***p****^agent^* (these are the first three parts of the environment state observation vector; see Equations (1) and (2)). The maximum number of steps allowed per single route (*N_stp.max_*) was set to 100 steps. Table 4 shows the values of the reward function parameters used in the experiments.

### 3.3. Simulation, Baseline-Method, and other Parameters

**Figure 5:**
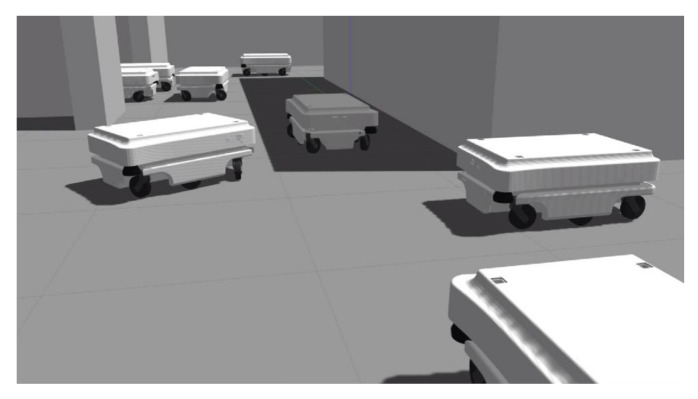
Physics-based simulation developed using Robot Operating System (ROS) and Gazebo simulator.

The physics-based simulation developed using Robot Operating System (ROS) and Gazebo simulator [21] was used to test the generated routes for feasibility and compare the proposed approach with the baseline method (Figure 5).

The proposed approach uses a simple PD line-following algorithm to traverse individual straight route segments. To determine their current position and orientation, the mobile robots in the proposed approach use the same localisation system as in the baseline method. Some simple traffic rules are used:

- Right-of-way rule (since there may still be some intersections);
- Leave enough space in front of the robot (stop and reverse very slowly if another robot is closer than 2.7 m in front of the robot on the same route segment);
- Stop (and reverse slowly) if the front of the mobile robot gets too close to another mobile robot).

For travel between pick-up and drop-off locations, the baseline approach uses a standard navigation system based on finding the shortest path to the goal location. In the baseline approach, the mobile robots move between pick-up and drop-off locations using the shortest paths calculated by the anytime A* algorithm.

**Figure 6:**
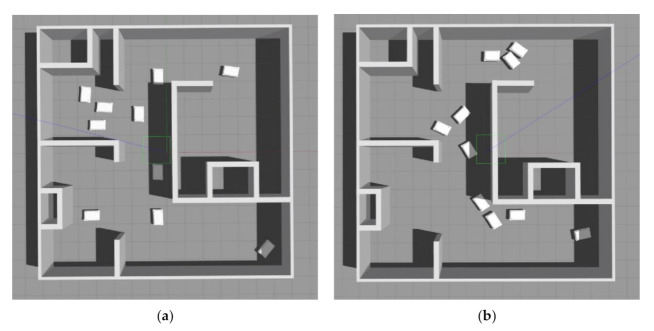
Simulation experiments for validation of the proposed method: (**a**) proposed approach; (**b**) baseline approach.

Figure 6 shows simulations using the proposed approach (a) and the baseline approach (b). With the proposed approach, the mobile robots are aligned in *x* and *y* directions since they use pre-computed routes that are all aligned in *x* and *y* directions (since the RL agent in the proposed method can only generate routes by moving up, down, left, or right). With the baseline approach, the robots use the shortest paths to travel between pick-up and drop-off locations; therefore, when there are a large number of mobile robots in a system, conflicts between the robots often occur.

### 3.4. Different Layouts

**Figure 7:**
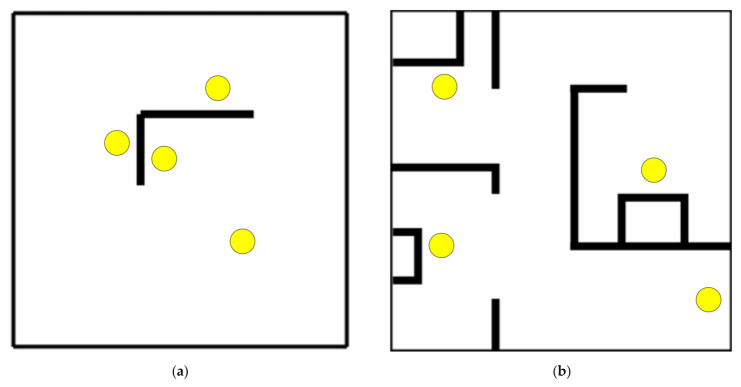

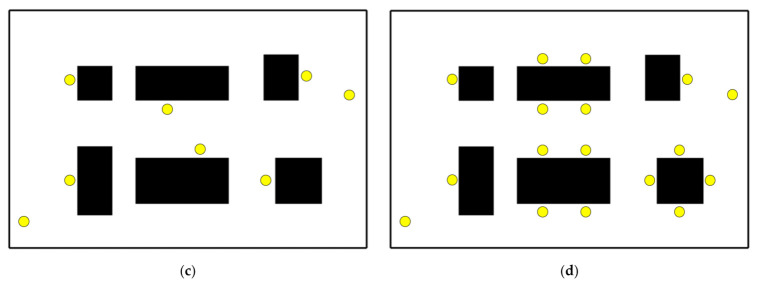
Other layouts: (**a**) four pick-up/drop-off locations, one wall, 12 × 12 m; (**b**) four pick-up/drop-off locations, multiple walls, 12 × 12 m; (**c**) eight pick-up/drop-off locations, 30 × 20 m; and (**d**) seventeen pick-up/drop-off locations, 30 × 20 m. Yellow circles are pick-up/drop-off locations, white represents the driving area, and black are the walls or other static obstacles.

The feasibility of the proposed approach is further demonstrated by applying it to four different layouts: (1) four pick-up/drop-off locations, one wall, 12 × 12 m (Figure 7a), (2) four pick-up/drop-off locations, multiple walls, 12 × 12 m (Figure 7b), (3) eight pick-up/drop-off locations, 30 × 20 m (Figure 7c), and (4) seventeen pick-up/drop-off locations, 30 × 20 m (Figure 7d). The cell size for all layouts is 1m. For layouts 1, 2, and 3 the total number of steps to train the reinforcement learning agent is set to 5 million. In the case of layout 4 (17 pick-up/drop-off locations, 30 × 20 m), the learning process is extended to 10 million steps.

## 4. Results

### 4.1. Generating Routes

**Figure 8:**
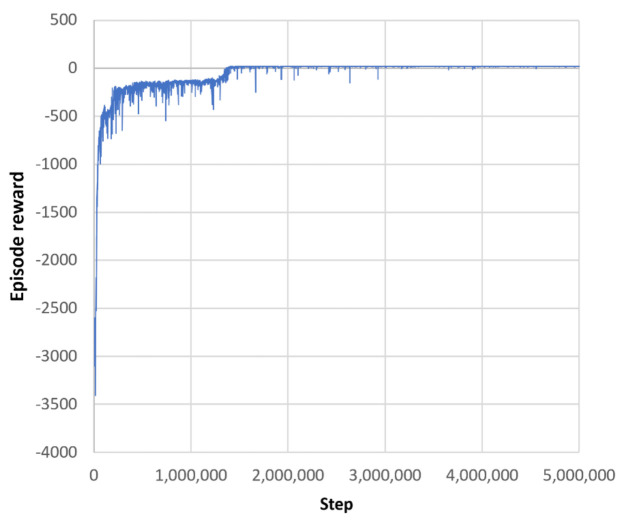
Episode reward during training the RL agent.

Figure 8 shows how the reinforcement-learning agent’s policy is improved over a learning process of 5 million steps.

**Figure 9:**
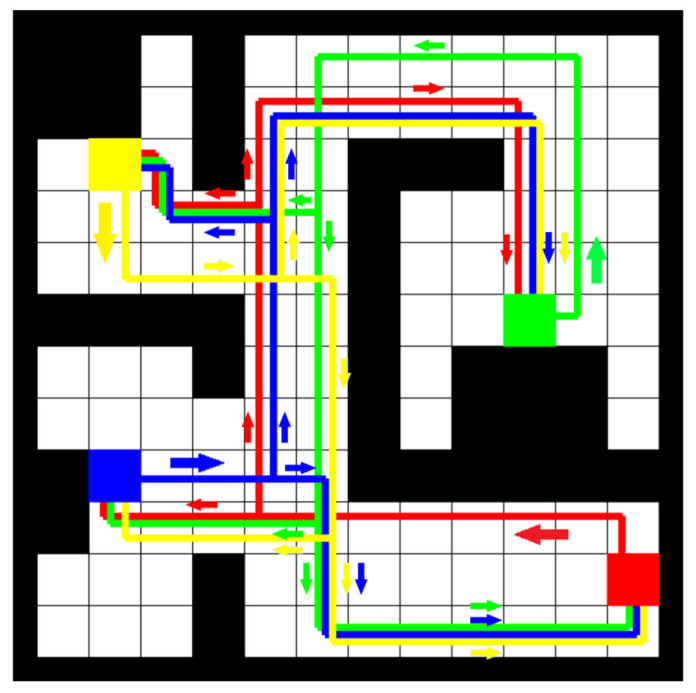
Routes generated by the trained RL agent. The coloured squares (blue, red, green, and yellow) indicate pick-up/drop-off locations, and the colour of the route indicates to which pick-up location the route belongs. All four locations (blue, red, green, and yellow squares) represent both pick-up as well as drop-off locations (robots may travel in both directions for any pair of these locations). When the robot needs to travel from a pick-up location to a drop-off location, it follows a route that is the same colour as the pick-up location.

The final routes generated by the trained reinforcement-learning agent are shown in Figure 9. The total reward for this episode was 17.88. The total time for learning the reinforcement learning agent and generating the final routes was 36 min.

### 4.2. Comparison of the Proposed to Baseline Approach

**Figure 10:**
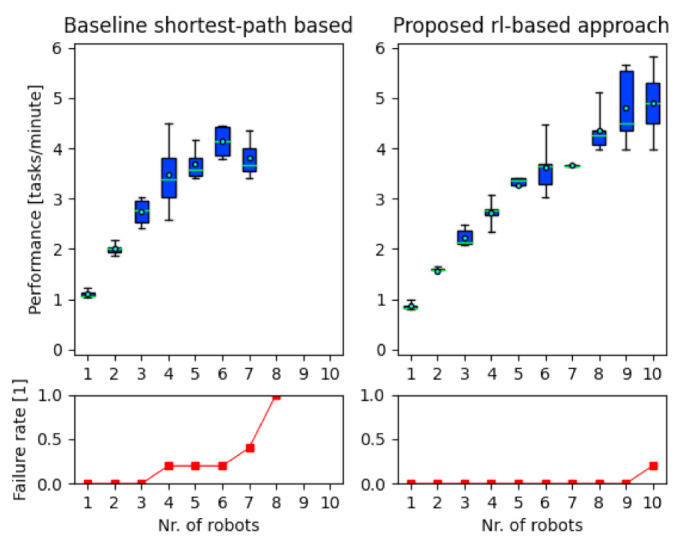
Performances of the proposed and the baseline approach. Above: the performances calculated according to Equation (5). Below: the proportion of tests in which a multi-AMR system failed to finish given tasks due to severe conflicts between the robots.

Figure 10 shows how the performance of a multi-robot logistics system changes as a function of the number of mobile robots operating in the system and depending on the navigation approach used. To calculate the average performance shown in the upper graphs in Figure 10, only the results of tests where the multi-robot system was able to complete all tasks were used. The lower graphs in Figure 10 show the fraction of tests in which a multi-robot system could not complete the given tasks due to severe conflicts/deadlocks between the robots. The failure rate is defined as the fraction of tests in which the AMR system failed to finish the given tasks *(failure_rate = nr_of_failed_examples/nr_of_all_tests*). Table 5 compares the route lengths of the shortest path based approach and the proposed approach.

### 4.3. Different Layouts

**Figure 11:**
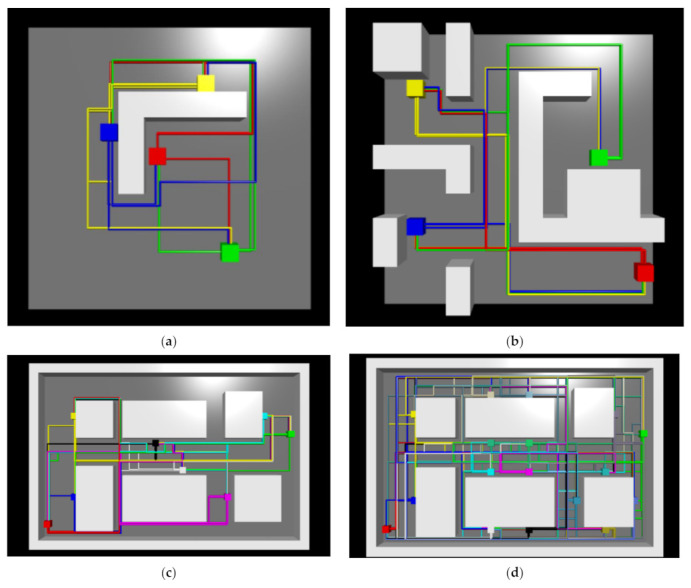
Results on different layouts: (**a**) four pick-up/drop-off locations, one wall, 12 × 12 m; (**b**) four pick-up/drop-off locations, multiple walls, 12 × 12 m; (**c**) eight pick-up/drop-off locations, 30 × 20 m; and (**d**) seventeen pick-up/drop-off locations, 30 × 20 m. The coloured squares (blue, red, green, and yellow, etc.) indicate pick-up/drop-off locations, the colour of the route indicates which pick-up location the route belongs to, the walls/obstacles are represented in light grey, and the driving surface is coloured dark grey.

**Figure 12:**
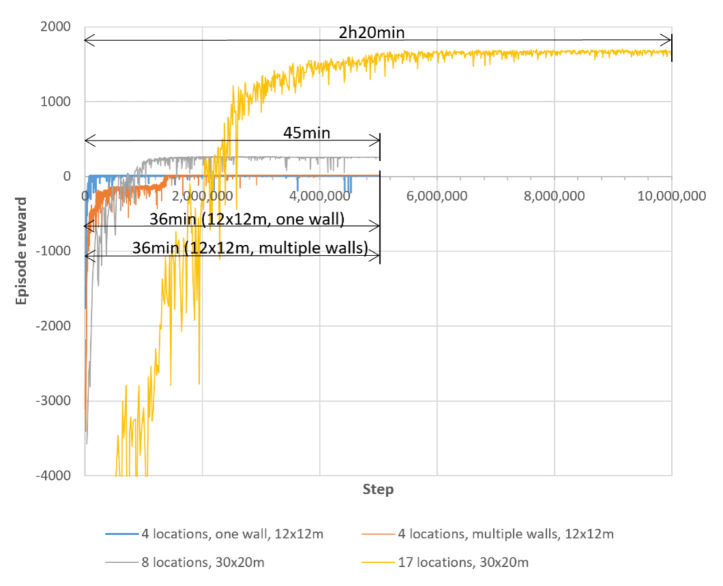
Episode reward during training and training times for different layouts. Tests were running on a mobile workstation with Intel Core i7–9750H Processor (12 × 2.6 GHz) and 32 GB RAM.

The proposed approach was able to find all routes in all four layouts (see Figure 11). Figure 12 shows the episode rewards during the learning process and the training times for each layout. Table 6 shows the average, minimum, and maximum route lengths. In the more complex last two layouts (Figure 11c,d), route segments occurred that went in opposite directions at the same locations. This occurred in three route segments (a total distance of 3 m) in the third layout (eight pick-up/drop-off locations, 30 × 20 m) and in eight route segments (a total distance of 8 m) in the fourth layout (seventeen pick-up/drop-off locations, 30 × 20 m).

## 5. Discussion

At the beginning of training, the agent starts learning from scratch and receives a low reward of about −3000 (see Figure 8). Most of the improvement is achieved within the first 1.5 million steps, during which the episode reward improves to almost 17. Over the next 3.5 million steps, the agent’s policy is fine-tuned and stabilised. From about 3.2 million steps onward, the episode reward remains at about 17.8.

With the proposed approach, it took 36 min to train the RL agent on a given layout and generate the routes. With the baseline approach, the time to compute the route between the pick-up and drop-off locations is for the same layout approximately one second, which means that the total time to compute all 12 routes is about 10 s. Route calculation time using the baseline approach generally depends on the size and complexity of the layout and the location and the distances between the start and destination locations. For larger and more complex layouts, the time to calculate the route will be longer on average. However, it is important to note that the baseline approach does not consider the multi-robot problem and when using the proposed approach, the computation of the routes is only required once at the beginning and is only repeated when the layout changes. In the usual implementation of the baseline approach, the paths are computed online, meaning that a new path is calculated every time a robot receives a new task or a goal destination.

As expected, the shortest-path-based approach produced shorter routes (Table 5). All lengths (min, max, and mean) are approximately 30% longer when using the proposed approach.

From the generated routes to be used in the multi-AMR system (Figure 9), it can be observed that there is no part of the route that would be in the same location (in the same cell) directed in the opposite direction to another existing part of the routes. The reason for this is probably a relatively large penalty if the RL agent takes a step to a location that is in the opposite direction to another existing part of the route. In the final route map, there are still intersections of routes because the solution does not exist without intersections for that layout. In contrast, sometimes it is better to create intersecting routes instead of very long routes without intersections. It can also be observed that two-lane roads are created where one lane has routes in one direction and the other lane has routes in the opposite direction. It seems that despite trying to follow all other rules (avoiding conflicts of opposite directions, intersections, etc.), the RL agent has made the routes as short as possible.

The experiment confirmed the hypothesis that in the presence of a large number of mobile robots, the use of the proposed approach allows the multi-AMR system to perform better than the commonly used baseline approach, which focuses only on calculating the shortest paths between pick-up and drop-off locations. The proposed approach performs better than the baseline approach on both criteria (throughput and scalability) in all cases where there are eight or more mobile robots in the system. The performance is not only better in terms of how many tasks are executed in a given time; such an approach is also more reliable as there was rarely any system failure (an exception was in one of the tests when all ten mobile robots were operating in the system). The proposed approach has 73.3% higher performance when only cases where no failures occurred are considered (in this case, the highest average performance of the baseline approach is when three mobile robots are operating in the system; in this case, the performance is 2.72 tasks/minute, in the proposed reinforcement learning based approach the best average performance is 4.72 tasks/minute when nine mobile robots are in the system). If the cases where the failures occurred are ignored, the average performance of the proposed approach is 16.8% higher (4.12 tasks/minute in the baseline approach when there are six robots in the system, and 4.81 tasks/minute in the proposed approach in the case of all ten mobile robots in the system).

**Figure 13:**
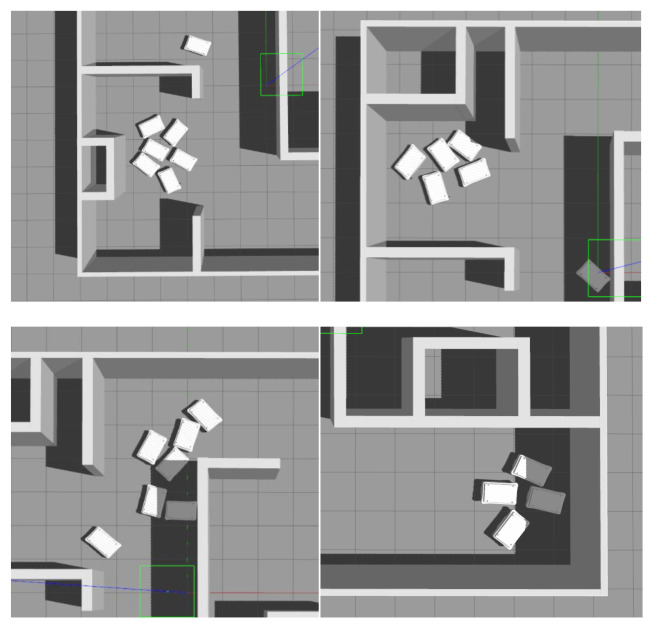
Typical failures in the shortest-path based (baseline) approach when having a large number of AMRs in the system.

The baseline approach failed to complete a single schedule with eight mobile robots in the system, and system failures occurred with only four mobile robots. In contrast, the proposed reinforcement learning-based approach succeeded in completing all but one of the 50 tests (5 different schedules; 10 different numbers of mobile robots). The system failed only in one case where all ten mobile robots were in the system, and this was due to suboptimal settings of the meeting rule parameters, which could be further improved relatively easily in the case of the proposed approach. In the baseline approach, in almost all cases of failure, situations occurred where multiple mobile robots travelling in opposite directions met head-on at a narrow passage or got stuck at pick-up/drop-off locations, causing a traffic jam that was impossible to get out of using the standard navigation method. Some of these failures are shown in Figure 13.

**Figure 14:**
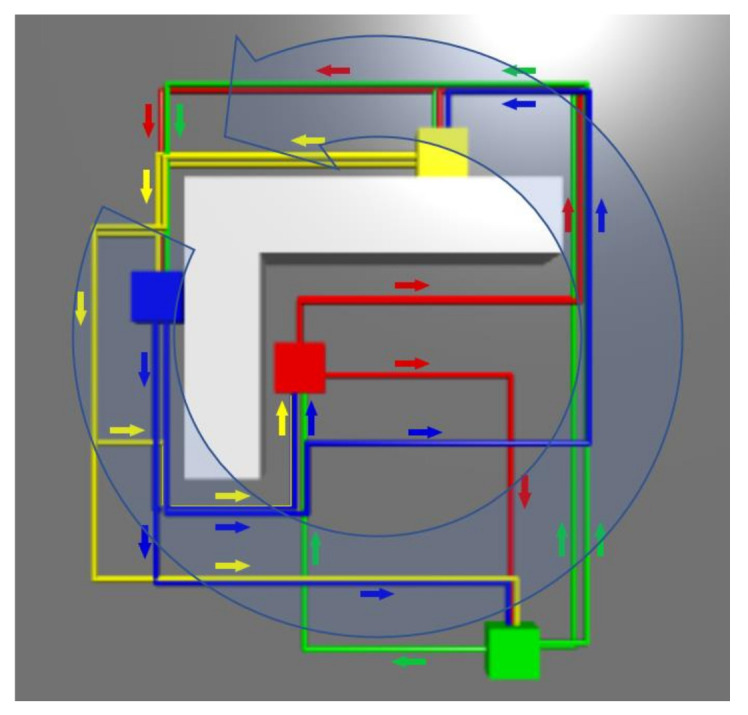
Circular traffic in a counter-clockwise direction around the wall.

The ability to generate routes using the proposed approach was also tested on different layouts (see Section 4.3). The proposed approach was able to find all routes in all tested layouts. The solution for the first layout (Figure 11a) seems interesting. In this case (four pick-up/drop-off locations, one wall, 12 × 12 m), the RL agent learns that the best solution is to set up counter-clockwise circular traffic around the wall in the middle of the room, as shown in Figure 14. The second layout (four pick-up/drop-off locations, multiple walls, 12 × 12 m) is the one we used in the previous section for comparison with the shortest path based approach. In the two more complex layouts with a larger number of pick-up/drop-off locations and a larger size of the driving area (see Figure 11c,d), some route segments were generated that went in opposite directions at the same location, but this occurred only to a very small extent (in 3 and 8 route segments, respectively). Due to the very infrequent occurrence, the route segments going in opposite directions can be identified and manually corrected, or additional driving rules can be added instead in the parts of the routes where traffic is running in both directions. One way to avoid this problem could also be to change the reward function (e.g., increase the penalty for counter-flowing routes in the same locations, in which case it might be worthwhile for the agent to find a different route, either longer or with more intersections, but without parts where the routes run in opposite directions).

In Figure 12, which shows the episode rewards during training and the training times for all four layouts, one can observe that the computational complexity most likely increases with the number of routes to be found. In particular, comparing the third (eight pick-up/drop-off locations, 30 × 20 m) and fourth (seventeen pick-up/drop-off locations, 30 × 20 m) layouts, it can be seen that for the same size and shape of the driving area, the convergence of the episode reward slows down with a larger number of pick-up/drop-off locations. Comparing the first (four pick-up/drop-off locations, one wall, 12 × 12 m) and second (four pick-up/drop-off locations, multiple walls, 12 × 12 m) layouts, which have the same number of pick-up/drop-off locations and approximately the same size of the driving area but a different configuration of walls, it can be seen that convergence in RL agent learning is also affected by the complexity of the obstacle configuration.

The advantages of the proposed method are:

- automatic generation of routes;
- scalability in terms of the number of mobile robots (the number of vehicles in the system does not affect the difficulty and time of calculating routes/actions);
- in the generation of routes, the relative importance of each criterion can be adjusted for the specific application of the proposed method;
- a good system performance can be achieved with only a few simple traffic rules;
- the use of the proposed navigation method allows a more accurate (and easier) prediction of the future states of mobile robots (this property could also be exploited in the extension of the method by adding the functionality offered by state-of-the-art on-line path planning methods);
- a small number of possible ways how mobile robots can meet (e.g., at an intersection, on a straight line, or at a turn), this facilitates increasing the robustness of the system;
- additional communication between the robots is minimal (the robots only need to share their current location and orientation);
- compared to the baseline approach, the use of computational resources is minimal (in addition to following the route, the robot uses a simple algorithm to check only the current geometric relations with other mobile robots, i.e., relative position and orientation);
- the proposed method allows for a relatively simple decentralisation of a multi-AMR system.

Potential advantages not currently included in the experiment, although the proposed method allows their realisation, are:

- the relative importance of each route can be adjusted (some routes may be more important or frequent than others);
- the relative importance of each robot or task could be set by dynamically adjusting the traffic rules;

The disadvantages are:

- the difficulty and time of route computation depends on the number of routes to be found;
- pick-up/drop-off locations must be determined in advance (e.g., if a pick-up/drop-off location later appears elsewhere, the default navigation system must be used to go to that location);
- routes found are static.

## 6. Conclusions

A method for automatically generating routes for multi-AMR systems is proposed. The proposed method, which generates routes between pick-up and drop-off locations, is based on reinforcement learning. With simulation experiments, it has been confirmed that using the proposed method with a larger number of mobile robots in the system can enable higher performance in terms of throughput and reliability. The main advantages of the method are the automatic generation of routes, the ability to consider several criteria simultaneously, the scalability in terms of the number of mobile robots, and the low load on the computational resources and the communication network. The disadvantages of the method are that the computational complexity increases with the number of routes, the pick-up, and drop-off locations must be determined in advance and the static nature of the generated routes. Given the common situations in today’s logistics systems that involve the use of AMRs, the static nature of the generated routes does not limit the feasibility of the proposed method for the majority of use cases.

## Figures and Tables

**Table 1:** The total number of different combinations of routes (middle column), and estimated time to find all combinations and to calculate the optimization function values for all combinations of routes (right column).

Example Layout	Total Nr. of Combinations of Routes	Estimated Time *
2 × 2 (Figure 2a)	4096	≈1 ms
3 × 3 (Figure 2b)	4.444945756 × 10^12^	≈13 years
4 × 4 (Figure 2c)	1.15513119 × 10^27^	≈4.4 × 10^15^ years

* The estimated times based on simulations running on a mobile workstation with Intel Core i7-9750H Processor (12 × 2.6 GHz) and 32 GB RAM.

**Table 2:** Values for the individual parts of the reward function.

Variable	Case 1	Value in Case 1	Case 2	Value in Case 2
*r_step_*	In each new step.	*R_step_* *	/	/
*r_sp_*	Shortest path length decreased.	*R_sp_*	Shortest path length increased.	*−R_sp_*
*r_position_*	Agent hits the wall or other static obstacle, or at the wrong drop-off location.	−1	Otherwise.	0
*r_visited_*	At the already visited location in current episode.	*R_visited_*	Otherwise.	0
*r_visit.c.r._*	At the already visited location, it was in searching for the current route.	−1	Otherwise.	0
*r_opp_*	The just-generated part of the route is directed in the opposite direction as another part of the already existing routes in the current agent’s position.	−1	Otherwise.	0
*r_cross_*	Crossing another existing route.	*n_cross_* · *R_cross_*	Otherwise.	0
*r_turn_*	Making a turn.	*R_turn_*	Otherwise.	0
*r_max.stp._*	The number of steps for current route exceeded the predefined maximum allowed number of steps for single route. (*n_stp.c.r._ > N_stp.max_*)	−1	Otherwise.	0
*r_dropoff_*	The agent arrives to drop-off location of current route.	1	Otherwise.	0

* The value ranges of certain parameters in this table can be found in Table 3. The settings of these parameters depend on a specific application case.

**Table 3:** Reward function parameters value ranges.

Value Range
−1 ≪ *R_step_* < 0
0 < *R_sp_* ≪ 1
−1 ≪ *R_visited_* < 0
−1 ≪ *R_cross_* < 0
−1 ≪ *R_turn_* < 0

**Table 4:** Reward function parameters.

Parameter	Value
*R_step_*	−0.001
*R_sp_*	0.05
*R_visited_*	−0.005
*R_cross_*	−0.05
*R_turn_*	−0.005

**Table 5:** Routes lengths, shortest path-based, and proposed approach.

Method	Min. Route Length (m)	Max. Route Length (m)	Route Length Mean ± st. dev. (m)
shortest-path-based	9.02	20.44	13.84 ± 3.91
proposed (RL-based)	12.00	29.00	18.83 ± 5.31

**Table 6:** Routes lengths, total number of routes and opposite route segments for different layouts.

Layout	Total Nr. of Routes	Min. Route Length [m]	Max. Route Length [m]	Route Length Mean ± st.dev. [m]	Nr. of Opposite Route Segments *
four pick-up/drop-off locations, one wall, 12 × 12 m	12	6.00	18.00	12.17 ± 4.84	0
four pick-up/drop-off locations, multiple walls, 12 × 12 m	12	12.00	29.00	18.83 ± 5.31	0
eight pick-up/drop-off locations, 30 × 20 m	56	5.00	48.00	25.07 ± 11.17	3
seventeen pick-up/drop-off locations, 30 × 20 m	272	3.00	66.00	24.57 ± 10.83	8

* one segment length is 1 m.
